# Primary care utilization for patients with newly diagnosed cancer during the COVID-19 pandemic: a population-based study

**DOI:** 10.1186/s12885-022-10257-4

**Published:** 2022-11-04

**Authors:** Ying Ling, Matthew C. Cheung, Kelvin K.W. Chan, Aisha Lofters, Colleen Fox, Aditi Patrikar, Ning Liu, Simron Singh

**Affiliations:** 1 Division of Hematology, Department of Medicine, University of Toronto, Toronto, Canada; 2 Odette Cancer Centre, Sunnybrook Health Sciences Centre, Toronto, Canada; 3 ICES, Toronto, Canada; 4 Division of Medical Oncology, Department of Medicine, University of Toronto, Toronto, Canada; 5 Institute of Health Policy, Management and Evaluation, University of Toronto, Toronto, Canada; 6 Department of Family and Community Medicine, University of Toronto, Toronto, Canada; 7 Peter Gilgan Centre for Women’s Cancers, Women’s College Hospital, Toronto, Canada; 8 Person-Centered Care, Ontario Health, Toronto, Canada

**Keywords:** Primary care, Cancer, COVID-19 pandemic, Virtual care

## Abstract

**Background:**

The COVID-19 pandemic greatly impacted primary care and cancer care. We studied how primary care utilization in Ontario, Canada changed for patients who were newly diagnosed with cancer just prior to the COVID-19 pandemic compared to those diagnosed in non-pandemic years.

**Methods:**

This population-based, retrospective cohort study used linked healthcare databases to compare outcomes for patients with a new malignancy diagnosed within the year prior to the COVID-19 pandemic, between July 1 and September 30, 2019 (COVID-19 cohort) to those diagnosed in the same months in 2018 and 2017 (pre-pandemic cohort). We used Poisson regression models to compare rates of in-person and virtual visits to patients’ usual primary care physician (PCP), emergency department (ED) visits, and hospitalizations, all reported per person-year of follow-up.

**Results:**

In-person visits to usual PCPs decreased from 4.07/person-year in the pre-pandemic cohort to 2.58 in the COVID-19 cohort (*p* < 0.0001). Virtual visits to usual PCPs increased from 0.00 to 1.53 (*p* < 0.0001). Combined in-person and virtual visits to patients’ usual PCPs was unchanged from 4.07 to 4.12 (*p* = 0.89). The rate of ED visits decreased from 0.99/person-year to 0.88 (*p* < 0.0001). Non-elective hospitalizations remained unchanged, from 0.49/person-year to 0.47 (*p* = 0.1675).

**Conclusion:**

There was a sizeable shift in primary care visits for cancer patients from in-person to virtual during the pandemic, although there was no resultant increase in hospitalizations. This suggests that early in the pandemic, virtual care allowed for continuity in utilization of primary care, though further studies are required to confirm this persisted later in the pandemic.

**Supplementary Information:**

The online version contains supplementary material available at 10.1186/s12885-022-10257-4.

## Introduction

Primary care physicians (PCPs) form an important backbone of our healthcare system. They are often the first to detect a possible diagnosis of cancer, either through routine screening or symptom assessment. During cancer treatment, they continue to provide support through monitoring of comorbidities and non-cancer related medications. The COVID-19 pandemic rapidly changed the delivery of primary care. Compared to tertiary hospitals, primary care clinics had more initial uncertainty regarding personal protective equipment availability and infrastructure to develop safe physical distancing protocols for in-person visits. Additionally, many PCPs were called upon to provide support for COVID-19 testing sites, assessment centres, and long term care facilities [1]. In Ontario, Canada, similar to elsewhere in the world, there were large shifts from in-person care to virtual care for PCPs during the COVID-19 pandemic, often with little preparation [2].

Cancer care has also been greatly affected by the COVID-19 pandemic. In the United States, cancer-related patient encounters and screening decreased over 40% and 80% respectively in 2020 compared to 2019 [3]. In Ontario, Canada, the provincial cancer screening programs delivered 41% fewer screening tests in 2020 compared to 2019 [4]. A modelling study from the United Kingdom predicted increases in avoidable deaths resulting from delays in cancer diagnosis due to the COVID-19 pandemic [5]. However, the true impact of the pandemic on primary care utilization for cancer patients and the downstream clinical consequences remains unclear. We aimed to understand how primary care utilization in Ontario, Canada changed for patients who were newly diagnosed with cancer just prior to the COVID-19 pandemic compared to those diagnosed in non-pandemic years.

## Methods

### Study design and data sources

We undertook a population-based, retrospective cohort study using linked healthcare administrative databases held at ICES in Ontario, Canada. ICES is an independent, non-profit research institute whose legal status under Ontario’s health information privacy law allows it to collect and analyze healthcare and demographic data for health system evaluation and improvement. The use of these data for our project did not require review by the Research Ethics Board as it complied with Sect. 45 of Ontario’s *Personal Health Information Protection Act*. Permanent residents of Ontario receive full medical care coverage through the province’s universal and publicly funded insurance plan, the Ontario Health Insurance Plan (OHIP). All linked data for eligible individuals in our study were linked using unique encoded identifiers and analyzed at ICES (see Appendix for database details on ICES databases used).

### Study population

Patients aged between 18 and 100 years with a new breast, lung, colorectal, prostate, hematologic, or head and neck squamous cell malignancy diagnosed within the year prior to the COVID-19 pandemic, between July 1, 2019 and September 30, 2019 (COVID-19 cohort) were compared to patients diagnosed in years unaffected by the COVID-19 pandemic, between July 1, 2018 – September 30, 2018 and July 1, 2017 – September 30, 2017 (pre-pandemic cohort). These malignancies were selected as they are the most common cancers to affect the adult population. These dates were selected due to the Ontario Cancer Registry (OCR) only having malignancy diagnostic data updated to September 2019 at the time of study. Nonetheless, as patients were followed for 12 months after initial cancer diagnosis, in the COVID-19 cohort, this allowed for up to 7 months of follow-up data occurring during the COVID-19 pandemic. The months were kept constant in each cohort to control for seasonal variation. Patients were excluded if they had a prior diagnosis of cancer, had another cancer diagnosed within the follow-up period, did not have a valid healthcare number, and did not have a primary care visit in the 2 years prior to their cancer diagnosis.

### Outcomes and measures

The primary outcome was the rate of in-person and virtual visits to PCPs per person-year of follow-up. PCP visits were identified using OHIP outpatient visit billing codes linked to specialty codes for family medicine/general practice and community medicine. Virtual visits included telephone or video calls identified through temporary OHIP virtual care codes implemented during the pandemic and pre-existing telemedicine care codes in the historical periods. Codes used specifically for videoconferencing through the Ontario Telemedicine Network were also included (Supplementary Table 3). We also assessed the rate of in-person and virtual visits to a patient’s usual PCP, identified either through a patient being rostered to that PCP through a primary care enrollment model, or the bulk of the patient’s primary care visits in the prior 2 years to that PCP if a patient is not rostered.

Secondary outcomes included rate of in-person and virtual visits to a specialist, emergency department (ED) visits, and hospitalizations. Specialist visits were further stratified into all specialists, hematologists, medical oncologists, and radiation oncologists using OHIP specialty codes. Hospitalization data were collected for all acute-care hospitalizations and specifically non-elective hospitalizations. Due to an observation in cancer clinics during the COVID-19 pandemic that patients reported difficulty seeing their PCPs for non-cancer related prescription refills, we also assessed the rate of prescriptions written by specialists for medications typically provided by PCPs in patients aged 65 or older (anti-hypertensives, oral hypoglycemic agents and insulins, statins, and thyroid replacements as documented within the Ontario Drug Benefits Claims database, Supplementary Table 4).

### Statistical analysis

Primary and secondary outcomes, reported as rate of visits per person-year of follow-up, were calculated separately for the COVID-19 cohort and the pre-pandemic cohort by dividing the total number of visits by the total person-years of follow-up for each cohort. The 95% confidence intervals (CI) of rates were generated assuming number of visits following a Poisson distribution. We used Poisson regression models to compare rates of visits between the COVID-19 cohort and the pre-pandemic cohort. SAS 14.3 was used for the analysis.

To assess the impact of changes in primary care practice during the COVID-19 pandemic on ED visits and non-elective hospitalizations, we additionally conducted a nested case control study within the COVID-19 cohort to compare exposures in patients who had an ED visit or non-elective hospitalization during the pandemic to those who did not. Cases were patients who had a non-COVID related ED visit or non-elective hospitalization (identified if there was no positive COVID-19 test on the Ontario Laboratories Information System database between 1 month before to 1 week after), occurring between June 1, 2020 and their 12-month follow-up from initial cancer diagnosis. This period was selected to allow a 3-month lookback window for assessment of exposures occurring during the COVID-19 pandemic. The index date for cases was defined as their first non-COVID related ED visit or non-elective hospitalization. Controls, also taken from the COVID-19 cohort, did not have an ED visit or non-elective hospitalization between June 1, 2020 and their 12-month follow-up, and were 2:1 matched to cases on cancer type, age, sex, and co-morbidity index. The pseudo-index date for the controls was then assigned to equate the same time from cancer diagnosis to the index date for the matched case counterpart; those with pseudo-index dates falling before June 1, 2020 were removed from analysis. Exposures of interest were having an above median number of in-person and virtual visits to PCPs and specialists, having one or more prior ED visit or non-elective hospitalization (occurring from initial cancer diagnosis up to June 1, 2020), metastatic cancer stage, and exposure to chemotherapy and radiation therapy. Odds ratios were calculated using logistic regression.

## Results

We identified 17,545 patients diagnosed with a new breast, lung, colorectal, prostate, hematologic, or head and neck squamous cell malignancy during the COVID-19 cohort timeframe with 15,775 person-years of follow-up, and 36,362 patients diagnosed with a new malignancy during the pre-pandemic cohort timeframe with 31,696 person-years of follow-up. Table 1 summarizes baseline patient characteristics and Table 2 summarizes characteristics of the patients’ usual PCPs.

**Table 1:** Baseline patient characteristics

Characteristic	Pre-Pandemic Cohort (*n* = 36,362)	COVID-19 Cohort (*n* = 17,545)	Standardized Difference
**Sex**			
Female, *n* (%)	17,675 (48.6%)	8,886 (50.6%)	0.041
**Age at index date (cancer diagnosis)**			
Mean (SD)	59.01 (12.18)	58.54 (12.03)	0.039
Median (IQR)	59 (51–67)	59 (51–67)	0.038
**Cancer type**, ***n*(%)**			
Breast	8,580 (23.6%)	4,528 (25.8%)	0.051
Colorectal	5,698 (15.7%)	2,833 (16.1%)	0.013
Head and neck	1,646 (4.5%)	793 (4.5%)	0.00
Hematologic	5,977 (16.4%)	2,882 (16.4%)	0.00
Lung	6,770 (18.6%)	3,103 (17.7%)	0.024
Prostate	7,691 (21.2%)	3,406 (19.4%)	0.043
**Cancer stage**, ***n*(%)**			
Metastatic	6,001 (16.5%)	1,678 (9.6%)	0.21*
Non-metastatic	21,799 (59.9%)	5,839 (33.3%)	0.56*
Missing	8,562 (23.5%)	10,028 (57.2%)	0.73*
**Income quintile**, ***n*(%)**			
1	7,148 (19.7%)	3,395 (19.4%)	0.008
2	7,517 (20.7%)	3,527 (20.1%)	0.014
3	7,230 (19.9%)	3,515 (20.0%)	0.004
4	6,955 (19.1%)	3,429 (19.5%)	0.011
5	7,404 (20.4%)	3,627 (20.7%)	0.008
Missing	108 (0.3%)	52 (0.3%)	0.00
**ON-Marg material deprivation index**, ***n*(%)**			
1	7,810 (21.5%)	3,877 (22.1%)	0.013
2	7,383 (20.3%)	3,580 (20.4%)	0.009
3	7,063 (19.4%)	3,444 (19.6%)	0.003
4	7,011 (19.3%)	3,286 (18.7%)	0.017
5	6,807 (18.7%)	3,215 (18.3%)	0.011
Missing	288 (0.8%)	143 (0.8%)	0.01
**ON-Marg dependency index**, ***n*(%)**			
1	6,471 (17.8%)	3,284 (18.7%)	0.026
2	6,494 (17.9%)	3,127 (17.8%)	0.00
3	6,558 (18.0%)	3,251 (18.5%)	0.011
4	6,968 (19.2%)	3,242 (18.5%)	0.02
5	9,583 (26.4%)	4,498 (25.6%)	0.018
Missing	288 (0.8%)	143 (0.8%)	0.01
**ON-Marg ethnic concentration index**, ***n*(%)**			
1	7,543 (20.7%)	3,621 (20.6%)	0.008
2	7,108 (19.5%)	3,476 (19.8%)	0.005
3	6,822 (18.8%)	3,164 (18.0%)	0.015
4	6,887 (18.9%)	3,323 (18.9%)	0.001
5	7,714 (21.2%)	3,818 (21.8%)	0.015
Missing	288 (0.8%)	143 (0.8%)	0.01
**ON-Marg residential instability index**, ***n*(%)**			
1	6,557 (18.0%)	3,329 (19.0%)	0.014
2	6,779 (18.6%)	3,319 (18.9%)	0.005
3	7,146 (19.7%)	3,386 (19.3%)	0.003
4	6,982 (19.2%)	3,350 (19.1%)	0.006
5	8,610 (23.7%)	4,018 (22.9%)	0.018
Missing	288 (0.8%)	143 (0.8%)	0.01
**Collective Co-morbidity burden (ADGs)**, ***n*(%)**			
0–4 ADGs	9,531 (26.2%)	4,690 (26.7%)	0.012
5–9 ADGs	18,497 (50.9%)	8,998 (51.3%)	0.022
10 + ADGs	8,334 (22.9%)	3,857 (22.0%)	0.008
**Persons with medical conditions associated with frailty)**, ***n*(%)**			
No	33,574 (92.3%)	16,370 (93.3%)	0.038
Yes	2,788 (7.7%)	1,175 (6.7%)	0.038
**Resource utilization bands**, ***n*(%)**			
0	45 (0.1%)	16 (0.1%)	0.01
1	185 (0.5%)	110 (0.6%)	0.016
2	1,627 (4.5%)	824 (4.7%)	0.011
3	17,205 (47.3%)	8,500 (48.4%)	0.023
4	9,485 (26.1%)	4,591 (26.2%)	0.002
5	7,815 (21.5%)	3,504 (20.0%)	0.038

ON-Marg, Ontario Marginalization Index; ADGs, Aggregate Diagnosis Groups

* These larger SD differences were due to the OCR database having a higher proportion of missing staging data for patients in the COVID-19 cohort

**Table 2:** Baseline usual primary care physician characteristics

Characteristic	Pre-Pandemic Cohort (*n* = 36,362)	COVID-19 Cohort (*n* = 17,545)	Standardized Difference
**PCP sex**, ***n*(%)**			
Female	13,064 (35.9%)	6,737 (38.4%)	0.051
Male	23,101 (63.5%)	10,682 (60.9%)	0.054
Missing	197 (0.5%)	126 (0.7%)	0.022
**PCP age at index date**			
Mean (SD)	54.93 (11.39)	55.52 (11.01)	0.053
Median (IQR)	55 (47–63)	56 (48–63)	0.046
**PCP years since date of first certification at index date**			
Mean (SD)	24.31 (8.50)	25.37 (8.31)	0.13
Median (IQR)	23 (17–31)	24 (18–31)	0.13
**PCP country of medical school**, ***n*(%)**			
Canadian	22,793 (62.7%)	10,075 (57.4%)	0.11
Non-Canadian	10,139 (27.9%)	4,758 (27.1%)	0.01
Missing	3,430 (9.4%)	2,712 (15.5%)	0.18
**PCP patient enrollment model (PEM) type**, ***n*(%)**			
FHG/CCM	10,853 (29.8%)	4,995 (28.5%)	0.03
FHT	11,090 (30.5%)	5,476 (31.2%)	0.016
Non-FHT	12,158 (33.4%)	6,061 (34.5%)	0.022
TFFS	1,666 (4.6%)	766 (4.4%)	0.01
Other PEM	409 (1.1%)	170 (1.0%)	0.015
Not enough visits to attribute to usual PCP	181–185	77 (0.4%)	0.01
Missing	1–5	0 (0.0%)	0.01

PCP, primary care physician; FHG/CCM, enhanced fee-for-service through a Family Health Group or Comprehensive Care Model; FHT, blended capitation with an interprofessional Family Health Team; Non-FHT, blended capitation without an interprofessional Family Health Team; TFFS, total fee-for-service not in an enrollment model

### Primary care visits

**Fig. 1:**
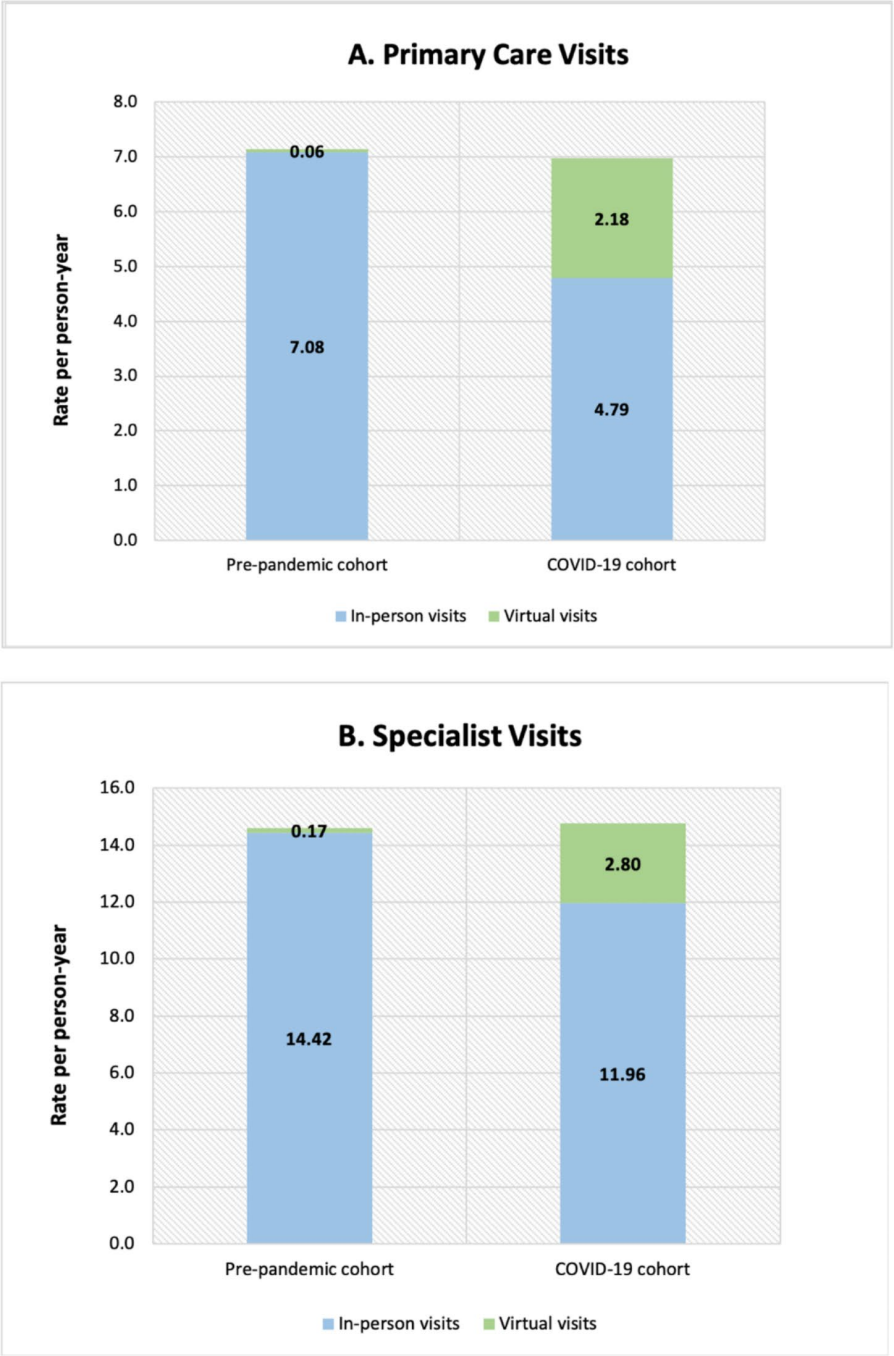
**A** Number of in-person and virtual visits per person-year to primary care physicians in the pre-pandemic and COVID-19 cohorts. **B** Number of in-person and virtual visits per person-year to specialist physicians in the pre-pandemic and COVID-19 cohorts

The rate of in-person visits to all PCPs decreased from 7.08 visits per person-year [95% CI 7.05–7.11] in the pre-pandemic cohort to 4.79 [4.76–4.82] in the COVID-19 cohort, corresponding to a 2.29 visits per person-year rate difference. Virtual visits to all PCPs per person-year increased from 0.06 [0.06–0.07] to 2.18 [2.16–2.20], an increase of 2.12 visits per person-year. When combining in-person and virtual visits to all PCPs, there was a decline from 7.14 [7.11–7.17] to 6.97 [6.93–7.01], a decrease of 0.17 visits per person-year (Fig. 1 A).

Similar findings were seen for rate of visits to usual PCPs. In-person visits to usual PCPs decreased from 4.07 visits per person-year [4.04–4.09] to 2.58 [2.56–2.61], a decrease of 1.49 visits per person-year, while virtual visits increased from 0.00 [0.00–0.01] to 1.53 [1.52–1.55], an increase of 1.53 visits per person-year. Combined in-person and virtual visits to usual PCPs did not change substantially, from 4.07 [4.05–4.09] to 4.12 [4.08–4.15].

### Specialist visits

The rate of in-person visits to all specialists decreased from 14.42 visits per person-year [14.38–14.46] in the pre-pandemic cohort to 11.96 [11.91–12.01] in the COVID-19 cohort, a decrease of 2.46 visits per person-year. Virtual visits to all specialists per person-year increased from 0.17 [0.17–0.18] to 2.80 [2.77–2.83], an increase of 2.63 visits per person-year. Combined in-person and virtual visits to all specialists increased from 14.60 [14.55–14.64] to 14.76 [14.70–14.82], an increase of 0.16 visits per person-year (Fig. 1B).

When stratifying visits specifically to oncologists, in-person visits decreased from 2.85 [2.83–2.86] to 2.30 [2.28–2.33] for medical oncologists, 1.10 [1.09–1.11] to 0.91 [0.89–0.92] for hematologists, and 2.86 [2.84–2.88] to 2.25 [2.22–2.27] for radiation oncologists, a decrease of 0.55, 0.19, and 0.61 visits per person-year respectively. Virtual visits to medical oncologists, hematologists and radiation oncologists increased from 0.06 [0.06–0.07] to 0.65 [0.64–0.67], 0.02 [0.02–0.02] to 0.18 [0.17–0.19], and 0.06 [0.05–0.06] to 0.53 [0.52–0.54], representing increases of 0.59, 0.16, and 0.47 visits per person-year respectively. Combined in-person and virtual visits to medical oncologists and hematologists did not change substantially, from 2.91 [2.89–2.93] to 2.96 [2.93–2.98] and from 1.11 [1.10–1.13] to 1.09 [1.07–1.10] respectively. Combined visits to radiation oncologists decreased from 2.92 [2.90–2.94] to 2.78 [2.75–2.80].

### Prescriptions for primary care medications

There was no significant change in the number of specialist prescriptions for medications typically prescribed by PCPs (Supplementary Table 4), from 0.37 [0.33–0.41] per person-year in the pre-pandemic cohort to 0.40 [0.35–0.46] in the COVID-19 cohort. Prescriptions for these medications written by PCPs also did not change substantially, from 2.56 [1.98–3.31] in the pre-pandemic cohort to 2.83 [2.00–4.00] in the COVID-19 cohort.

### ED visits and hospitalizations

The rate of visits to the ED decreased from 0.99 [0.98–1.00] per person-year in the pre-pandemic cohort to 0.88 [0.87–0.90] in the COVID-19 cohort, a decrease of 0.11 visits per person-year. Total hospitalizations decreased from 0.85 [0.84–0.86] to 0.81 [0.80–0.83], a decrease of 0.04 visits per person-year, but non-elective hospitalizations did not substantially change, from 0.49 [0.48–0.50] to 0.47 [0.46–0.49].

Within the COVID-19 cohort, we identified 2,460 patients who had an ED visit (cases) and 3,334 patients who did not have an ED visit (controls) occurring between June 1, 2020 and their 12-month follow-up. Exposures that increased odds for an ED visit included having above the median number of virtual visits to a PCP, in-person or virtual visits to a specialist, or chemotherapy in the 3-month lookback window, as well as metastatic cancer stage and 1 or more ED visit prior to June 1, 2020 (Table 3).

**Table 3:** Odds ratios for exposures within the COVID-19 cohort for patients who had compared to those who did not have an ED visit on or after June 1, 2020

Exposure (3-month lookback unless otherwise indicated)	Odds Ratio (95% CI)	*p* value
In-person visit to a PCP (above median)	1.09 (0.94–1.26)	0.26
Virtual visit to a PCP (above median)	2.35 (2.05–2.70)	< 0.0001
In-person visit to a specialist (above median)	1.32 (1.15–1.52)	0.0001
Virtual visit to a specialist (above median)	1.57 (1.22–2.01)	0.0004
Radiation	1.19 (0.95–1.50)	0.13
Chemotherapy	1.40 (1.21–1.62)	< 0.0001
1 or more prior ED visit (from time of initial cancer diagnosis up to June 1, 2020)	1.91 (1.68–2.17)	< 0.0001
Metastatic cancer stage (at diagnosis)	1.39 (1.10–1.76)	0.012

We identified 988 patients who had a non-elective hospitalization (cases) and 1,366 patients who did not have a non-elective hospitalization (controls) within the COVID-19 cohort occurring between June 1, 2020 and their 12-month follow-up. Exposures that increased odds for a non-elective hospitalization included increased number of in-person or virtual visits to a PCP, in-person visits to a specialist, or chemotherapy in the 3-month lookback window, as well as metastatic cancer and 1 or more non-elective hospitalizations prior to June 1, 2020 (Table 4).

**Table 4:** Odds ratios for exposures within the COVID-19 cohort for patients who had compared to those who did not have a non-elective hospitalization on or after June 1, 2020

Exposure (3-month lookback unless otherwise indicated)	Odds Ratio (95% CI)	*p* value
In-person visit to a PCP (above median)	1.36 (1.10–1.69)	0.0041
Virtual visit to a PCP (above median)	1.85 (1.49–2.30)	< 0.0001
In-person visit to a specialist (above median)	1.66 (1.34–2.07)	< 0.0001
Virtual visit to a specialist (above median)	1.12 (0.79–1.58)	0.54
Radiation	1.05 (0.74–1.49)	0.80
Chemotherapy	1.46 (1.16–1.83)	0.0012
1 or more prior non-elective hospitalization (from time of initial cancer diagnosis up to June 1, 2020)	2.00 (1.62–2.48)	< 0.0001
Metastatic cancer stage (at diagnosis)	2.00 (1.41–2.82)	0.0001

## Discussion

In this large, population-based, retrospective cohort study of patients with newly diagnosed malignancies, we describe a major shift in ambulatory practices during the COVID-19 pandemic from in-person to virtual care. In the primary care setting, there was a slight decrease in the rate of combined visits to all PCPs, though the absolute difference was small, and there was no significant difference in the rate of combined visits to patients’ usual PCPs. Combined visits to all specialists increased slightly during the pandemic, with no meaningful difference noted for visits specifically to hematologists or medical oncologists. The rate of ED visits and total hospitalizations decreased in the COVID-19 cohort, a phenomenon that has been well described during the early pandemic, likely due to multiple factors including patient concerns of COVID-19 exposure as well as stay-at-home orders [6–8]. However, we found no significant difference in the rate of non-elective hospitalizations between the two cohorts.

From the start of the pandemic, there has been substantial concern from both patients and practitioners on the impact of COVID-19 for cancer patients. A systematic review found 62 studies that identified 38 different categories of delays and disruptions to cancer services including impacts on diagnosis and treatment, though there was no specific comment on use of primary care [9]. In Ontario, Canada, many public health interventions were put into place that could have affected regular healthcare use for cancer patients [10]. A study of primary care practices in our province found that total primary care visits decreased between March and July, 2020, but the smallest declines occurred in patients with expected high healthcare resource use [2]. For newly diagnosed cancer patients, a study in the United States found a higher uptake of telemedicine use within 30 days of cancer diagnosis in those with the highest socioeconomic status, though the downstream impact of this disparity was unclear [11]. A separate small study comparing the outcomes of 206 cancer patients newly starting systemic therapy in 2020, where at least one visit was virtual, to 105 patients in 2019, where all visits were in-person, found no difference in time to staging imaging, time to therapy initiation, 3 month ED visits and hospitalizations, or treatment delays [12]. In our much larger, population-based cohort study, we similarly found that the rapid shift in ambulatory practices during the pandemic to virtual medicine, even in the critically important domain of primary practice, did not compromise the care of newly diagnosed cancer patients by increasing unplanned hospital admissions.

We found that the rate of prescriptions written by specialists for medications that are commonly prescribed by primary care did not change substantially during the pandemic. In addition, PCPs did not write fewer prescriptions for these medications. Taken together, despite the anecdotal concern that patients had more difficulty reaching their PCPs for prescription refills, these findings suggest that virtual care adequately adapted to support ongoing care of baseline comorbidities in patients with new malignancies, and that utilization of PCPs was maintained.

Among patients in the COVID-19 cohort, chemotherapy exposure within the prior 3 months and metastatic cancer stage at diagnosis increased odds for an ED visit or non-elective hospitalization during the pandemic period, likely reflecting the higher care needs of these populations. Although we also noted a higher risk of ED visit/hospitalization associated with preceding increased outpatient visits to specialists and PCPs, further studies are needed to determine whether this was due to PCPs and specialists appropriately identifying higher risk patients and seeing them more frequently.

Some limitations of our study, given its population-scale data, include inability to obtain more granular data on specific reasons for outpatient visits, ED visits, and hospitalizations. These data also do not include assessment of important patient reported measures such as preference, experience, or satisfaction with changes during the pandemic. Additionally, these data do not account for variation in practice across the province. Our study excluded those with a second malignancy diagnosed during follow-up (1,785 in the pre-pandemic and 978 in the pandemic cohort) to ensure we knew to which diagnosis a visit corresponded; therefore, results cannot be generalized to patients with more than one cancer. The ICES databases vary in their updating timelines, and at the time of developing our study, we were limited by the lack of malignancy diagnostic data in the OCR beyond September 2019, and as a result, some follow-up data for the pandemic cohort occurs pre-pandemic. Consequently, the reported rate differences in our study may not fully reflect changes in healthcare utilization before and during the pandemic. There was also delayed reporting of staging data, which limited our ability to properly match for or assess differences as a result of metastatic disease. However, since the diagnoses were made in July – September 2019, before cancer screening and diagnostic testing would have been affected by the pandemic, we would not expect to see large differences in proportion of more advanced disease between the two cohorts. We also do not have mature outcome data beyond 1 year after cancer diagnosis, and thus cannot generalize our findings to later waves of the pandemic or on longer term impacts on cancer patients with increased virtual care.

## Conclusion

To our knowledge, this is the first large population-based study to describe primary care utilization for patients with newly diagnosed cancer during the COVID-19 pandemic. We found that there was a sizeable shift in outpatient care from in-person to virtual, for both PCPs and specialists. There was no change in the ability of patients to visit their usual PCP during the pandemic, albeit in a different method. No difference was seen in downstream clinical outcomes such as obtaining prescriptions for common primary care medications and reassuringly, there was no change in non-elective hospitalizations. While these findings suggest that in the short term, virtual care has allowed for stable utilization of primary care for patients with cancer, further studies are required to confirm this persists into later parts of the pandemic, ideally including a focus on the patient experience.

## Electronic supplementary material

Below is the link to the electronic supplementary material.

Supplementary Material 1

## Data Availability

The dataset from this study is held securely in coded form at ICES. While data sharing agreements prohibit ICES from making the dataset publicly available, access may be granted to those who meet pre-specified criteria for confidential access, available at www.ices.on.ca/DAS. The full dataset creation plan and underlying analytic code are available from the authors upon request, understanding that the computer programs may rely upon coding templates or macros that are unique to ICES and are therefore either inaccessible or may require modification.

## List of abbreviations

PCP: Primary care physician
ED: Emergency department
OHIP: Ontario Health Insurance Plan
OCR: Ontario Cancer Registry
