# Supplementary material for: Primary care utilization for patients with newly diagnosed cancer during the COVID-19 pandemic: a population-based study
Source: BMC Cancer. 2022 Nov 4;22:1133. doi: 10.1186/s12885-022-10257-4 (PMC9636629; doi:10.1186/s12885-022-10257-4)
Supplement: Supplementary file 1 — Supplementary Material 1 [file 12885_2022_10257_MOESM1_ESM.docx]

**Appendix**

ICES Databases

Cancer type and stage at diagnosis for new breast, lung, colorectal, prostate, hematologic, or head and neck squamous cell malignancies were obtained from the Ontario Cancer Registry (OCR), a provincial-wide registry that captures all new cancer diagnoses and all cancer deaths in the province since 1964. Solid tumour diagnoses in the OCR follow the National Cancer Institute’s Surveillance, Epidemiology, and End Results coding rules while hematologic malignancies use codes from the International Classification of Diseases for Oncology, 3rd edition (Supplementary Tables 1 and 2).

Demographic data were obtained from the Registered Persons Database, which provides sex, age, and postal code of individuals with an Ontario Health Insurance Plan (OHIP) card. Using neighbourhood income data from Statistics Canada paired with postal codes, income quintile was determined with 1 being the lowest and 5 being the highest quintile. We used the Ontario Marginalization Index (ON-Marg), a multifaceted index that combines key demographic indicators, to measure four dimensions of marginalization in our study: dependency, material deprivation, ethnic concentration and residential instability.(12) Using a 2-year lookback window, the Johns Hopkins Adjusted Clinical Group® (ACG®) System (version 10) was applied to determine baseline co-morbidity burden using Aggregate Diagnosis Groups (ADGs), frailty using the Medically Frail Condition Marker, and expected healthcare use using Resource Utilization Bands with 0 being no health care use and 5 being the highest expected use.(13, 14)

Outpatient visits were identified using the OHIP database for physician claims and this was linked to the Corporate Provider Database (CPDB) and ICES Physician Database (IPDB) for physician specialty. PCP characteristics including sex, age, years since first certification, country of medical school, and primary care enrollment model were also collected from the IPDB.

Additional data were collected from the Canadian Institute of Health Information Discharge Abstract Database for hospitalizations, the National Ambulatory Care Reporting System for emergency department visits, and Ontario Drug Benefit Claims (ODB) for medication prescriptions in patients over the age of 65 years.

**Supplementary Tables and Figures**

*Supplementary Table 1: OCR codes used to define included lung, colorectal, breast, prostate, and head and neck malignancies*

| **Cancer Type** | **OCR Cancer Site Code** | **Cancer Site** |
| --- | --- | --- |
| **Lung** | C339 | Trachea |
|  | C340 | Main bronchus |
|  | C341 | Upper lobe, lung |
|  | C342 | Middle lobe, lung |
|  | C343 | Lower lobe, lung |
|  | C348 | Overlapping lesion of lung |
|  | C349 | Lung, NOS |
|  | C384 | Pleura, NOS |
| **Colorectal** | C180 | Cecum |
|  | C181 | Appendix |
|  | C182 | Ascending colon |
|  | C183 | Hepatic flexure of colon |
|  | C184 | Transverse colon |
|  | C185 | Splenic flexure of colon |
|  | C186 | Descending colon |
|  | C187 | Sigmoid colon |
|  | C188 | Overlapping lesion of colon |
|  | C189 | Colon, NOS |
|  | C199 | Rectosigmoid junction |
|  | C209 | Rectum, NOS |
|  | C210 | Anus, NOS |
|  | C211 | Anal canal |
|  | C212 | Cloacogenic zone |
|  | C218 | Overlapping lesion of rectum, anus and anal canal |
| **Breast** | C500 | Nipple |
|  | C501 | Central portion of breast |
|  | C502 | Upper-inner quadrant of breast |
|  | C503 | Lower-inner quadrant of breast |
|  | C504 | Upper-outer quadrant of breast |
|  | C505 | Lower-outer quadrant of breast |
|  | C506 | Axillary tail of breast |
|  | C508 | Overlapping lesion of breast |
|  | C509 | Breast, NOS |
| **Prostate** | C619 | Prostate gland |
| **Head and Neck** | C10.0, C10.1, C10.2, C10.3, C10.4, C10.8, C10.9 | Oropharynx |
|  | C01.9, C02.0, C02.1, C02.2, C02.3, C02.4, C02.8, C02.9 | Tongue |
|  | C03.0, C03.1, C03.9 | Gum |
|  | C04.0, C04.1, C04.8, C04.9 | Floor of mouth |
|  | C05.0, C05.1, C05.2, C05.8, C05.9 | Palate |
|  | C06.0, C06.1, C06.2, C06.8, C06.9 | Other parts of mouth |
|  | C07.9 | Parotid gland |
|  | C08.0, C08.1, C08.8, C08.9 | Salivary glands |
|  | C09.0, C09.1, 09.8, C09.9 | Tonsil |
|  | C11.0, C11.1, C11.2, C11.3, C11.8, C11.9 | Nasopharynx |
|  | C12.9 | Pyriform Sinus |
|  | C14.0, C14.2, C14.8 | Other Sites in lip, oral cavity, and pharynx |
|  | C76.0 | Other ill-defined head, face or neck |
|  | C06.9, C14.8 | Oral Cavity |
|  | C32.0, C32.1, C32.3, C32.8, C32.9 | Larynx |
|  | C13.0, C13.1, C13.2, C13.8, C13.9 | Hypopharynx |
|  | C00.0, C00.1, C00.2, C00.3, C00.4, C00.5, C00.6, C00.8, C00.9, C14.8, C44.0 | Lip |

*Supplementary Table 2: OCR morphology codes used to define included hematologic malignancies*

| **Hematology Malignancy Type** | **OCR Morphology Code** | **Morphology Description** |
| --- | --- | --- |
| **Aggressive Lymphomas** | 95903 | Malignant lymphoma, NOS |
|  | 95913 | Malignant lymphoma, non-Hodgkin, NOS |
|  | 95963 | Composite Hodgkin and non-Hodgkin lymphoma |
|  | 96503 | Hodgkin lymphoma, NOS |
|  | 96513 | Hodgkin lymphoma, lymphocyte-rich |
|  | 96523 | Hodgkin lymphoma, mixed cellularity, NOS |
|  | 96533 | Hodgkin lymphoma, lymphocyte depletion, NOS |
|  | 96543 | Hodgkin lymphoma, lymphocyte depletion, diffuse fibrosis |
|  | 96553 | Hodgkin lymphoma, lymphocyte depletion, reticular |
|  | 96593 | Hodgkin lymphoma, nodular lymphocyte predominance |
|  | 96613 | Hodgkin granuloma |
|  | 96623 | Hodgkin sarcoma |
|  | 96633 | Hodgkin lymphoma, nodular sclerosis, NOS |
|  | 96643 | Hodgkin lymphoma, nodular sclerosis, cellular phase |
|  | 96653 | Hodgkin lymphoma, nodular sclerosis, grade 1 |
|  | 96673 | Hodgkin lymphoma, nodular sclerosis, grade 2 |
|  | 96733 | Mantle cell lymphoma |
|  | 96753 | Malignant lymphoma, mixed small and large cell, diffuse |
|  | 96783 | Primary effusion lymphoma |
|  | 96793 | Mediastinal large B-cell lymphoma |
|  | 96803 | Malignant lymphoma, large B-cell, diffuse, NOS |
|  | 96843 | Malignant lymphoma, large B-cell, diffuse, immunoblastic, NOS |
|  | 96873 | Burkitt lymphoma, NOS |
|  | 97023 | Mature T-cell lymphoma, NOS |
|  | 97053 | Angioimmunoblastic T-cell lymphoma |
|  | 97083 | Subcutaneous panniculitis-like T-cell lymphoma |
|  | 97143 | Anaplastic large cell lymphoma, T cell and Null cell type |
|  | 97163 | Hepatosplenic (gamma-delta) cell lymphoma |
|  | 97173 | Intestinal T-cell lymphoma |
|  | 97193 | NK/T-cell lymphoma, nasal and nasal-type |
|  | 98273 | Adult T-cell leukemia/lymphoma (HTLV-1 positive) |
|  | 98323 | Prolymphocytic leukemia, NOS |
|  | 98263 | Burkitt cell leukemia |
|  | 98333 | Prolymphocytic leukemia, B-cell type |
|  | 98343 | Prolymphocytic leukemia, T-cell type |
| **Indolent Lymphomas** | 96893 | Splenic marginal zone B-cell lymphoma |
|  | 96903 | Follicular lymphoma, NOS |
|  | 96913 | Follicular lymphoma, grade 2 |
|  | 96953 | Follicular lymphoma, grade 1 |
|  | 96983 | Follicular lymphoma, grade 3 |
|  | 96993 | Marginal zone B-cell lymphoma, NOS |
|  | 97003 | Mycosis fungoides |
|  | 97013 | Sezary syndrome |
|  | 96703 | Malignant lymphoma, small B lymphocytic, NOS |
|  | 96713 | Malignant lymphoma, lymphoplasmacytic |
|  | 97093 | Cutaneous T-cell lymphoma, NOS |
|  | 97183 | Primary cutaneous CD30+ T-cell lymphoproliferative disorder |
|  | 97613 | Waldenstrom macroglobulinemia |
|  | 98233 | B-cell chronic lymphocytic leukemia/small lymphocytic lymphoma |
|  | 99403 | Hairy cell leukemia |
| **Acute Leukemias** | 97273 | Precursor cell lymphoblastic lymphoma, NOS |
|  | 97283 | Precursor B-cell lymphoblastic lymphoma |
|  | 97293 | Precursor T-cell lymphoblastic lymphoma |
|  | 98373 | Precursor T-cell lymphoblastic leukemia |
|  | 98603 | Myeloid leukemia, NOS |
|  | 98613 | Acute myeloid leukemia, NOS |
|  | 97423 | Mast cell leukemia |
|  | 98003 | Leukemia, NOS |
|  | 98013 | Acute leukemia, NOS |
|  | 98053 | Acute biphenotypic leukemia |
|  | 98203 | Lymphoid leukemia, NOS |
|  | 98353 | Precursor cell lymphoblastic leukemia, NOS |
|  | 98363 | Precursor B-cell lymphoblastic leukemia |
|  | 98403 | Acute myeloid leukemia, M6 type |
|  | 98663 | Acute promyelocytic leukemia |
|  | 98673 | Acute myelomonocytic leukemia |
|  | 98703 | Acute basophilic leukemia |
|  | 98713 | Acute myeloid leukemia with abnormal marrow eosinophils |
|  | 98723 | Acute myeloid leukemia, minimal differentiation |
|  | 98733 | Acute myeloid leukemia without maturation |
|  | 98743 | Acute myeloid leukemia with maturation |
|  | 98763 | Atypical chronic myeloid leukemia, BCR/ABL negative |
|  | 98913 | Acute monocytic leukemia |
|  | 98953 | Acute myeloid leukemia with multilineage dysplasia |
|  | 98963 | Acute myeloid leukemia, t(8;21)(q22;q22) |
|  | 98973 | Acute myeloid leukemia, 11q23 abnormalities |
|  | 99103 | Acute megakaryoblastic leukemia |
|  | 99203 | Therapy-related acute myeloid leukemia, NOS |
|  | 99483 | Aggressive NK-cell leukemia |
| **Other** | 99453 | Chronic myelomonocytic leukemia, NOS |
|  | 99463 | Juvenile myelomonocytic leukemia |
|  | 98633 | Chronic myeloid leukemia, NOS |
|  | 98753 | Chronic myelogenous leukemia, BCR/ABL positive |
|  | 97333 | Plasma cell leukemia |
|  | 97313 | Plasmacytoma, NOS |
|  | 97323 | Multiple myeloma |
|  | 97343 | Plasmacytoma, extramedullary (not occurring in bone) |

*Supplementary Table 3: OHIP billing codes for virtual and telemedicine visits**

| **Fee Code** | **Description** | **Effective Dates** | **Payment** |
| --- | --- | --- | --- |
| **K080** | Minor assessment of a patient by telephone or video, or advice or information by telephone or video to a patient’s representative regarding health maintenance, diagnosis, treatment and/or prognosis | March 14, 2020 onwards | $23.75 |
| **K081** | Intermediate assessment of a patient by telephone or video, or advice or information by telephone or video to a patient’s representative regarding health maintenance, diagnosis, treatment and/or prognosis, if the service lasts at least 10 minutes; or psychotherapy, psychiatric or primary mental health care, counseling, or interview by telephone or video, if the service lasts at least 10 minutes | March 14, 2020 onwards | $36.85 |
| **K082** | Psychotherapy, psychiatric or primary mental health care, counseling, or interview by telephone or video per unit (‘unit’ means half-hour or major part thereof) per unit | March 14, 2020 onwards | $67.75 |
| **K083** | Specialist consultation or visit by telephone or video payable in increments of | March 14, 2020 onwards | $5.00 per increment |
| **B103A** | Hosted video visit: a synchronous video visit with a patient who is physically located and supported at a patient host site during the clinical encounter. Host sites are secure physical environments that organizations offer on-site to provide patients with convenient access to videoconferencing technology and, in some cases, clinical support services (nursing support, diagnostics through peripheral devices) | April 1, 2020 onwards | $0.00 (tracking code) |
| **B203A** | Direct-to-patient video visit: a synchronous video visit with a patient in the home or another location of their choice (i.e. the patient is not at a patient host site). This includes situations where a patient is scheduling and managing the encounter independently using their own technology, or where an organization is providing support resources that are with the patient (e.g. nursing support, technology) | April 1, 2020 onwards | $0.00 (tracking code) |
| **B099A** | Direct-to-patient video visit (see above) | November 15, 2019 to March 31, 2020 | $0.00 (tracking code) |
| **B100A** | Hosted video visit (see above), first telemedicine patient encounter premium | March 31, 2020 and prior | $35.00 |
| **B200A** | Hosted video visit (see above), subsequent telemedicine patient encounter premium | March 31, 2020 and prior | $15.00 |
| **B101A** | Hosted video visit (see above), first cancelled/missed telemedicine patient encounter premium | March 31, 2020 and prior | $35.00 |
| **B201A** | Hosted video visit (see above), subsequent cancelled/missed telemedicine patient encounter premium | March 31, 2020 and prior | $15.00 |
| **B102A** | Hosted video visit (see above), first technical difficulties abandoned patient encounter premium | March 31, 2020 and prior | $35.00 |
| **B202A** | Hosted video visit (see above), subsequent technical difficulties abandoned patient encounter premium | March 31, 2020 and prior | $15.00 |

**please see these links for further details:* [*https://www.health.gov.on.ca/en/pro/programs/ohip/bulletins/4000/bul4745.aspx*](https://www.health.gov.on.ca/en/pro/programs/ohip/bulletins/4000/bul4745.aspx)

[*https://www.health.gov.on.ca/en/pro/programs/ohip/bulletins/4000/bul4750.aspx*](https://www.health.gov.on.ca/en/pro/programs/ohip/bulletins/4000/bul4750.aspx)

[*https://www.health.gov.on.ca/en/pro/programs/ohip/bulletins/4000/bul4731.aspx*](https://www.health.gov.on.ca/en/pro/programs/ohip/bulletins/4000/bul4731.aspx)

*Supplementary Table 4: Medications included in analysis of prescriptions commonly written by PCPs*

| **Anti-Hypertensives** | | |
| --- | --- | --- |
| Acebutolol  Amlodipine*  Atenolol*  Benazepril  Bisoprolol  Candesartan*  Captopril  Carvedilol  Clorthalidone*  Cilazapril*  Diltiazem  Enalapril  Eplerenone  Eprosartan | Felodipine  Fosinopril  Furosemide  Hydralazine  Hydrochlorothiazide*  Indapamide*  Irbesartan*  Labetalol  Lisinopril*  Losartan*  Metoprolol  Nifedipine  Nimodipine  Olmesartan* | Perindopril*  Propanolol  Quinapril*  Ramipril*  Spironolactone*  Telmisartan*  Trandolapril  Valsartan*  Verapamil |
| **Thyroid Replacements** | **Hypoglycemic Agents** | **Statins** |
| Liothyronine  Levothyroxine | Acarbose  Canagliflozin  Dapagliflozin*  Empagliflozin*  Gliclazide  Glyburide  Insulins  Linagliptin*  Metformin*  Pioglitazone  Repaglinide  Rosiglitazone  Saxagliptin*  Sitagliptin* | Atorvastatin*  Fluvastatin  Lovastatin  Pravastatin  Rosuvastatin  Simvastatin |

* Combination drugs with these medications were also included
